# Specific microstructural changes of the cervical spinal cord in syringomyelia estimated by diffusion tensor imaging

**DOI:** 10.1038/s41598-021-84164-2

**Published:** 2021-03-04

**Authors:** Weifei Wu, Xiangxiang Li, Zong Yang, Neng Ru, Fan Zhang, Jie Liang, Ke Zhang

**Affiliations:** 1 Department of Orthopedics, The People’s Hospital of China Three Gorges University, The First People’s Hospital of Yichang, Yichang, Hubei China; 2 Department of Orthopedics, The People’s Hospital of WuFeng, Yichang, Hubei China

**Keywords:** Neuroscience, Diseases, Medical research, Neurology

## Abstract

The microstructure of the spinal cord in syringomyelia has not been well studied. The aim of this study was to evaluate the microstructure of the cervical cord in patients with syringomyelia using diffusion tensor imaging (DTI) and to investigate the association between DTI parameters and the size of the syrinx cavity. Thirty patients with syringomyelia and 11 age-matched controls were included in this study. DTI and T1/T2-weighted MRI were used to estimate spinal microstructure. The patients were divided into a clinical symptom group (group A) and a non-clinical symptom group (group B) according to ASIA assessments. The fractional anisotropy (FA) and apparent diffusion coefficient (ADC) values (mm^2^/s) were measured and compared between patients and controls. Correlation between FA/ADC and the size of the syrinx cavity was examined with a bivariate analysis. FA values were lower (*P* < 0.000) and ADC values were higher (*P* < 0.000) compared to the controls at the level of all syrinxes examined in patients with syringomyelia; both FA values and ADC values reached normal values either above or below the syrinx levels (all *P* > 0.05). FA values and ADC values at all cervical levels were not significantly different either in controls or outside of the syrinx (all *P* > 0.05). FA values of group A was significantly lower than those of group B (*P* < 0.000). There was a negative association between FA values and the size of syrinx cavity, and a positive association between ADC values and the size of syrinx cavity (FA: *P* < 0.05, ADC: *P* < 0.05). The microstructure of the cervical spinal cord is different across all patients with syringomyelia. DTI is a promising tool for estimating quantitative pathological characteristics that are not visible with general MRI.

## Introduction

Syringomyelia is a chronic disease of the spinal cord caused by fluid-filled cavities that form a lesion in the spinal cord, which induces gradual constriction of the spinal cord parenchyma followed by clinical symptom including spasticity and sensory disturbances^1,2^. Conventional magnetic resonance imaging (MRI) is currently the most widely used technique for diagnosing syringomyelia using T2-weighted imaging, which often displays anomalous signal intensity. However, conventional MRI, such as T1- and T2-weighted imaging, can only provide limited macroscopic information, including oedema-like changes and haemorrhage. In addition, conventional MRI findings do not correlate with a diagnosis based on clinical abnormalities^3^. Either the cavity in spinal cord is much larger than the clinical manifestation that would be expected or the other way around.

Diffusion tensor imaging (DTI) has been used widely to assess diseases of central nervous system and peripheral nervous system^4–7^. The parameters commonly used to delineate the microarchitecture of the spinal cord include fractional anisotropy (FA) and apparent diffusion coefficient (ADC), which reflect the direction and speed of the movement of water molecules^8^. At the lesion or the compressed level in patients with cervical spondylotic myelopathy, when compared to healthy control participants FA values were significantly reduced while ADC values were significantly increased^7,9^. However, there was no notable difference in DTI values between the non-compressed spinal cord of patients and healthy volunteers^9^. In patients with syringomyelia, ADC values were significantly higher than those of controls, but FA values were statistically lower than those of normal controls. Moreover, outside the visible syrinx cavity, the mean FA values were not decreased^10,11^.

Although several studies have reported using DTI in patients with syringomyelia, the length of syrinx cavity which may include several segments was regarded as a whole when calculated DTI values in previous studies. No study has previously reported whether the size of the syrinx is associated with FA/ADC values. The purpose of this study was to assess the association between DTI parameters and the size of the syrinx cavity and to investigate differences between patients with syringomyelia and controls at different cervical levels.

## Materials and methods

### Subjects

Thirty inpatients and outpatients with syringomyelia were prospectively studied from July 2016 to April 2019. There were 20 females and 10 males, and the mean age was 19 years (range 10–29 years). All patients were diagnosed with syringomyelia with or without Chiari malformation based on MRI findings. The patients were divided into a clinical symptom group (group A) and a non-clinical symptom group (group B). Based on the American Spinal Injury Association (ASIA) assessment, patients in Group A had nerve function at levels A, B, C and D, and patients in Group B had nerve function at level E^12^. Patients that had previous spinal surgery, tumour, trauma, or a secondary syrinx cavity were excluded from this study. The control group was composed of age-matched healthy participants without nerve symptoms and with a normal spinal cord, which was determined with MRI scanning; this group included four males and seven females with a mean age of 17 years (age range 10–22 years). This study was approved by the review board of the China Three Gorges University. All study methods were conducted in accordance with the China Three Gorges University guidelines and regulations. In addition, all experimental protocols were approved by the China Three Gorges University committee. Informed consent was obtained from a parent or legal guardian of participants to allow their clinical data to be used for research.

### MRI and DTI scanning

All imaging was performed on a 1.5 T scanner (GE Healthcare, United States) equipped with a multichannel all-spine coil. For conventional sequences, the scanning orientations used were sagittal T1-weighted images, sagittal T2-weighted images and transverse T2-weighted images. The images in this study were obtained with a field of view (FOV) of 250 mm for sagittal scanning and 230 mm for transverse scanning; the image matrix was 256 × 256. Parameters for the DTI sequence were the following: slice thickness = 2.5 mm; slice gap = 0 mm, 128 × 128 reconstruction matrix size, FOV = 230 × 230 mm. Diffusion was measured along six non-collinear directions with two b values (0, 1000 s/mm^−2^).

### Imaging process

**Figure 1:**
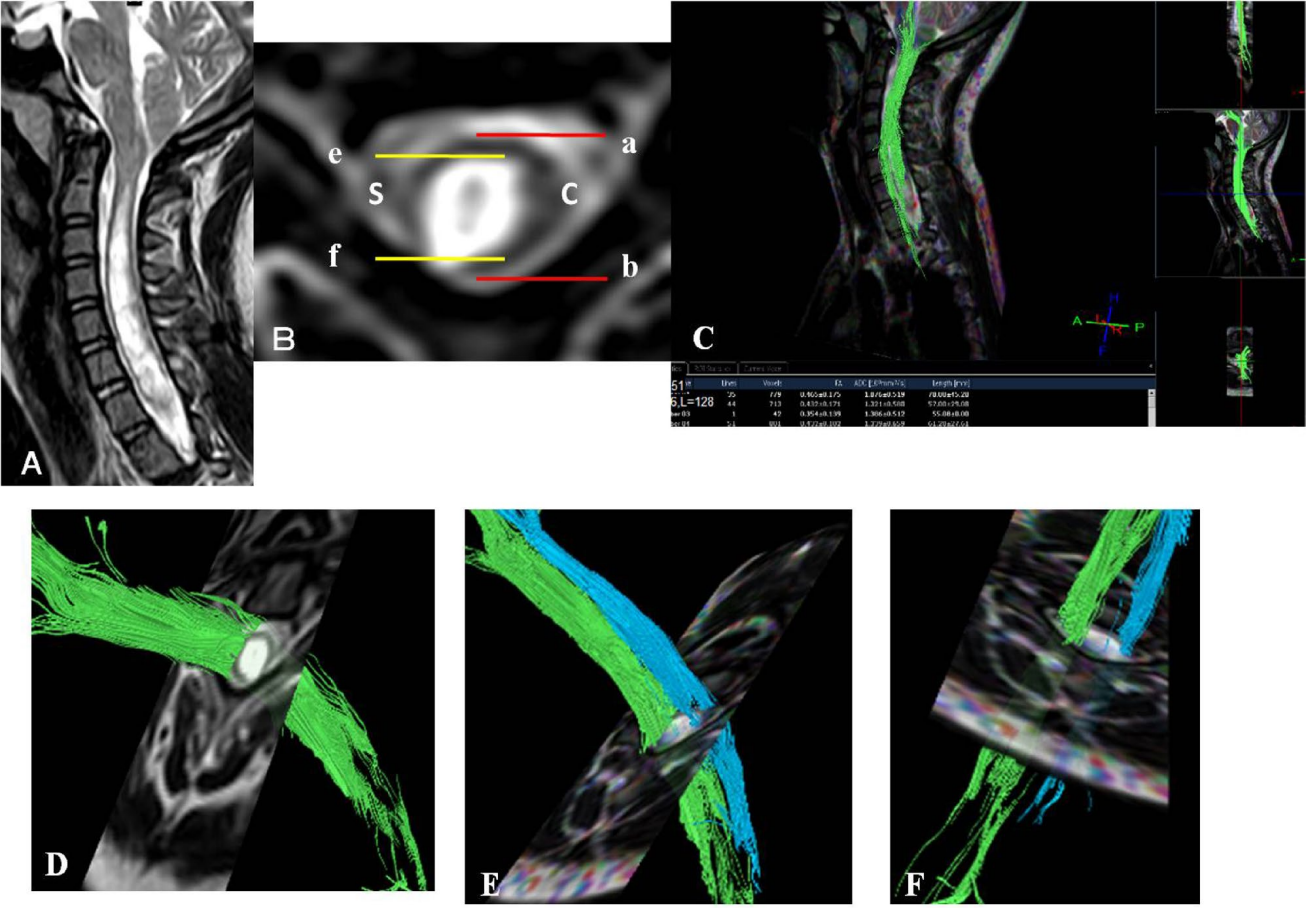
(**A**) MRI image at sagittal plane on T2W, (**B**) the ratio as index of syrinx dimension which was calculated by maximal syrinx diameter (S)/spinal cord diameter (**C**) at the same location on T2W axial. (**C**) Location map and measurement of DTI parameters on sagittal plane. (**D**–**F**) Measurement of DTI parameters on transverse plane.

The diffusion tensor images were transferred to the workstation and analysed by two experienced neuro-radiologists who were blinded to patients’ clinical data. Disagreements were resolved via discussion until decisions were reached by consensus. The evaluation focused on the DTI and conventional MRI analysis and was combined with the radiographic findings. FA and ADC maps were calculated using DTI image reconstruction software. The ADC and FA values were measured by defining regions of interest (ROIs, all pixels) in the cervical spinal cord of all participants at seven different segments: C1, C2, C3, C4, C5, C6 and C7 (Fig. 1). ROIs were selected with a fixed position using T2-weighted MRI on transverse planes and sagittal planes. Six ROIs were placed at the margin of the spinal cord, and two ROIs were placed at the centre of the grey matter of the spinal cord. The median level of the intervertebral space at T2-weighted MRI sagittal plane was as the ROIs of the axial plane. The intervals between the adjacent ROIs in the white matter were 60°, and the six ROIs were 360° in the axial plane of the spinal cord. The syrinx cavity was defined as the hyperintensity on a T2-weighted image. The normal cord segment for patients with syringomyelia was defined as the cranial or caudal segments proximal to the syrinx cavity. The ratio as well as the maximal syrinx diameter/spinal cord diameter at the same level used to measure DTI parameters on T2W axial was measured as an index of the syrinx dimension (Fig. 1).

### Statistical analysis

The statistical analysis was performed using a standard SPSS16.0 (SPSS Institute, Chicago, IL) software package. Data was expressed with mean ± SD A bivariate analysis of correlation was performed between FA/ADC and size of syrinx cavity. Several one-way ANOVAs were carried out to compare the following: the syrinx cavity of patients, normal spinal cord of patients and normal spinal cord of controls at the same segment; different segments in the control group; and group A, group B and healthy controls. Pearson’s correlation coefficients were used to determine the association between the size of the syrinx cavity and DTI metrics. *P* < 0.05 was considered statistically significant.

### Ethics approval and consent to participate

The review boards of the China Three Gorges University approved the present study. All participators signed informed consent of permitting their clinical data to be used for the study.

### Consent for publication

The authors report no conflict of interest concerning the materials or methods used in this study or findings specified in this paper. The authors have reviewed the final version of the manuscript and approve it for publication.

## Results

**Figure 2:**
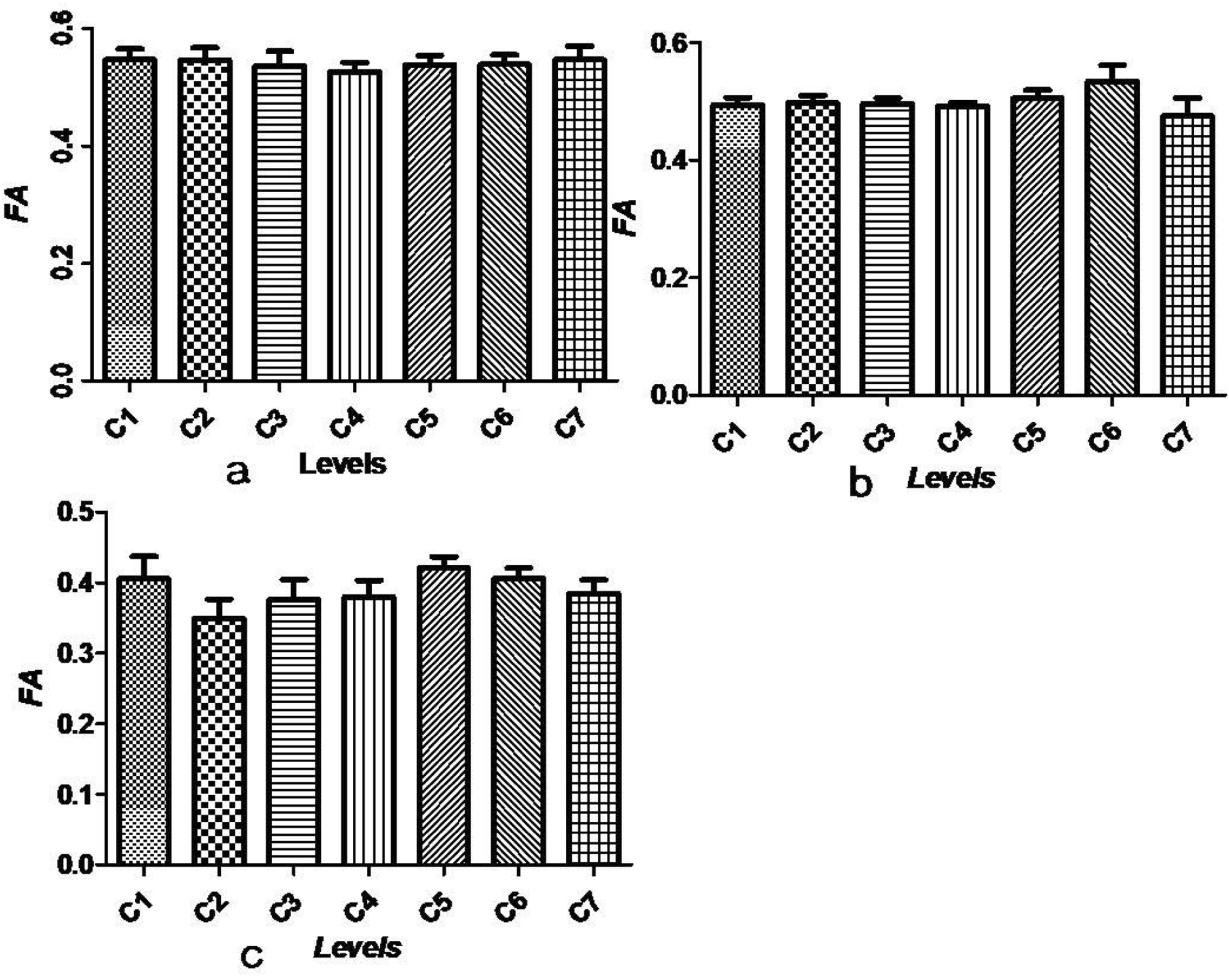
(**a**–**c**) FA values (mean ± SEM) at all cervical levels in controls, outside the area of the visible syrinx and the area of the visible syrinx, respectively.

**Figure 3:**
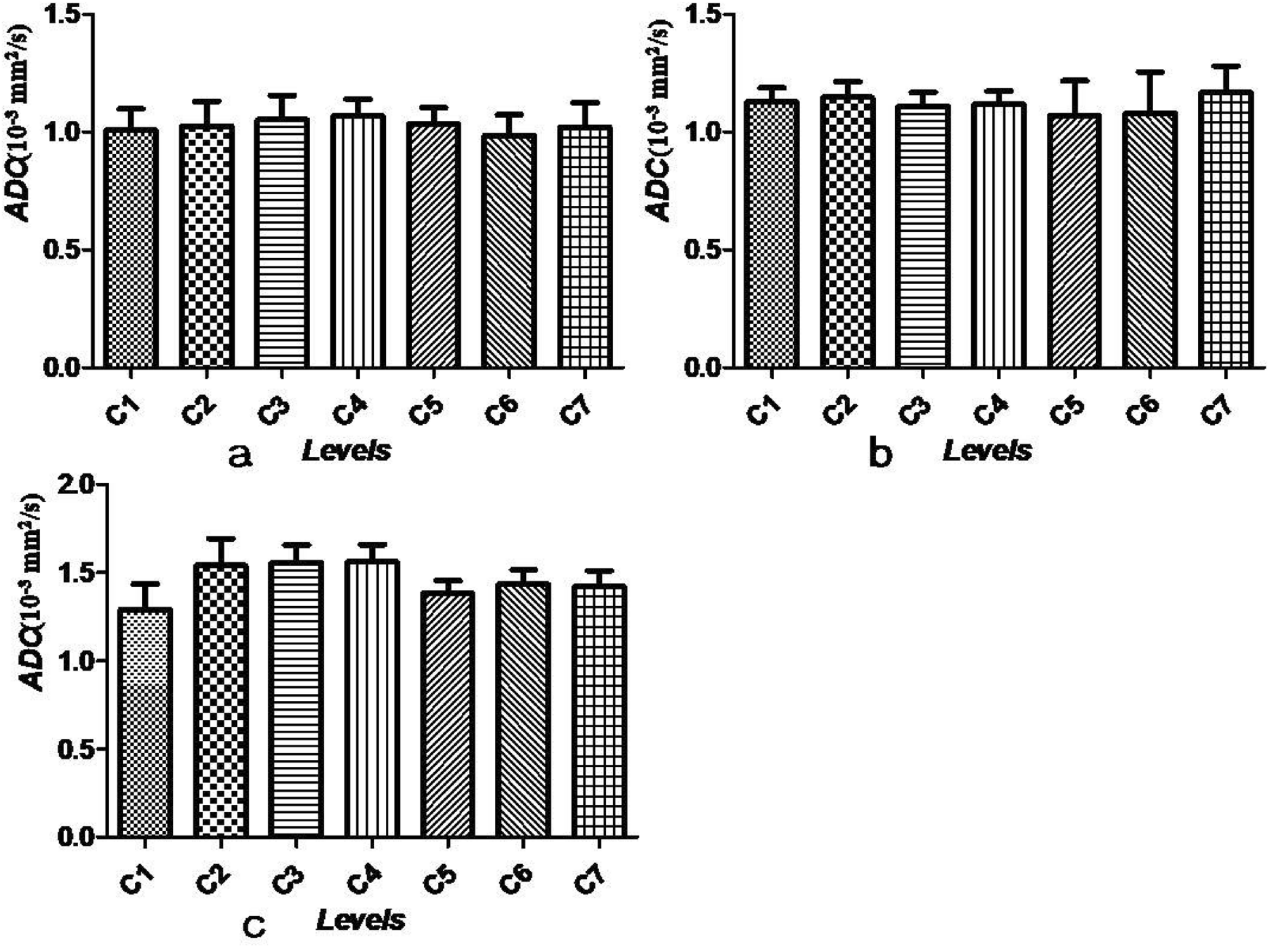
(**a**–**c**) ADC values (mm^2^/s: mean ± SEM) at all cervical levels in controls, outside the area of the visible syrinx and the area of the visible syrinx, respectively.

Detailed distribution information of syrinxes is listed in Table 1. The syrinx locations in the patients included locations at C5–T2 (two patients), C5–C6 (three patients), C5–C7 (five patients), below T2 (three patients), the entire spinal cord (two patients) and a different level (one patient). In the control group, the highest FA value (0.55) and ADC value (1.07 mm^2^/s) of the cervical cord segment were located at C7 and C4, respectively. The lowest FA and ADC values (0.53, 0.99 mm^2^/s) were located at C4 and C6, respectively (Figs. 2a, 3a). A one-way ANOVA examining seven different segments of the cervical spinal cord using DTI images showed that there was no difference among FA values (*P* = 0.987) or ADC values (*P* = 0.997).

**Table 1:** The distribution range of syrinx cavity and the number of patients.

Segment location	Number
C4–T1	1
C4–T5	1
C5–C6	3
C6–T8	1
C5/C6	1/1
C5–T2	1
C1–C6	1
C5–T2	2
C5–C7	5
C2–T10	1
Below T2	3
Entire spinal cord	2
C2–L5	1
C5–T12	1
C1–T5	1
C3–T5	1
C3–T3	1
C6–C7	1
C2–C7	1

Outside the visible syrinx cavity in patients with syringomyelia, the mean FA levels were 0.49 (C1), 0.50 (C2), 0.50 (C3), 0.49 (C4), 0.51 (C5), 0.53 (C6) and 0.47 (C7). The mean ADC values were 1.13 (C1), 1.15 (C2), 1.11 (C3), 1.12 (C4), 1.07 (C5), 1.08 (C6) and 1.17 (C7). No significant differences among the seven segments were found either for FA values or ADC values (*F* = 0.623, *P* = 0.712) (Figs. 2b, 3b). In the syrinx cavity, the mean FA values were 0.41 (C1), 0.35 (C2), 0.38 (C3), 0.38 (C4), 0.42 (C5), 0.41 (C6) and 0.39 (C7). The mean ADC values were 1.29 (C1), 1.54 (C2), 1.56 (C3), 1.56 (C4), 1.38 (C5), 1.44 (C6) and 1.42 (C7) (Figs. 2c, 3c). At the syrinx cavity, the mean FA values were significantly lower at every segment of the cervical spinal cord compared with the control group values and outside the area of the visible syrinx (all *P* < 0.05). The mean ADC values of the syrinx cavity from C1 to C7 were notably higher compared with those in the control group (all *P* < 0.05). Although the mean ADC values at C6 and C7 in the syrinx cavity were higher than those outside the visible syrinx, no statistical differences between the two groups were found (all *P* > 0.05). At every segment from C1 to C7, neither a decrease in the mean FA values nor an increase in the mean ADC values were found outside the area of the visible syrinx when compared with those in control group (all *P* > 0.05).

**Figure 4:**
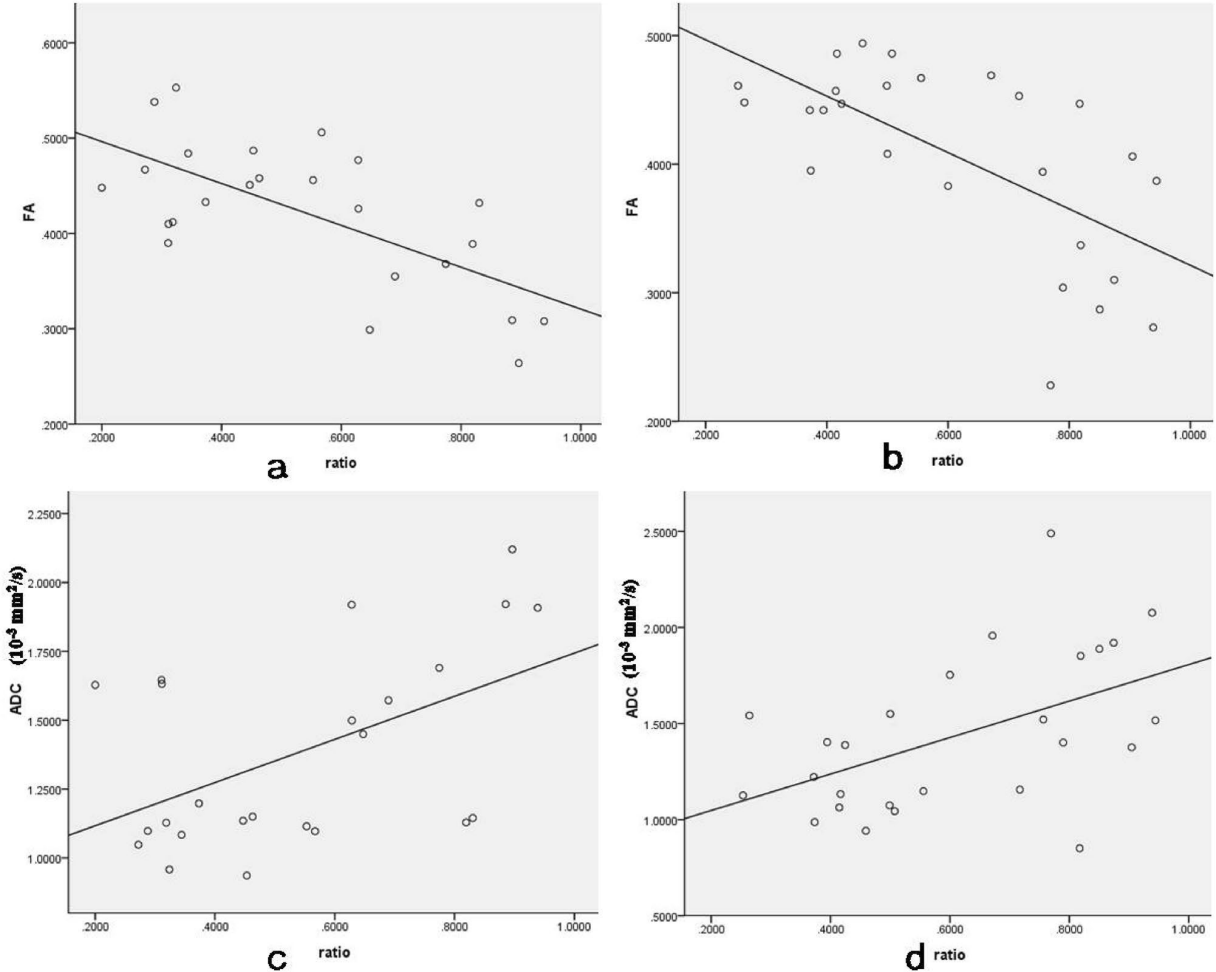
The association between size of syrinx cavity and FA values at C5 (**a**) and C6 (**b**) was negative. However, about ADC values (mm^2^/s) the association was positive (**c**,**d**).

Due to the number of samples were small, we only performed correlative analyses at C5 and C6. The association between the size of the syrinx cavity and FA values was significantly negative at both C5 and C6 (r-value: − 0.669 [C5], − 0.649 [C6], *P* < 0.05) (Fig. 4a,b). However, the association between size of syrinx cavity and ADC values was notably positive for both C5 and C6 (r-value: 0.508 [C5], 0.587 [C6], *P* < 0.05) (Fig. 4c,d).

**Figure 5:**
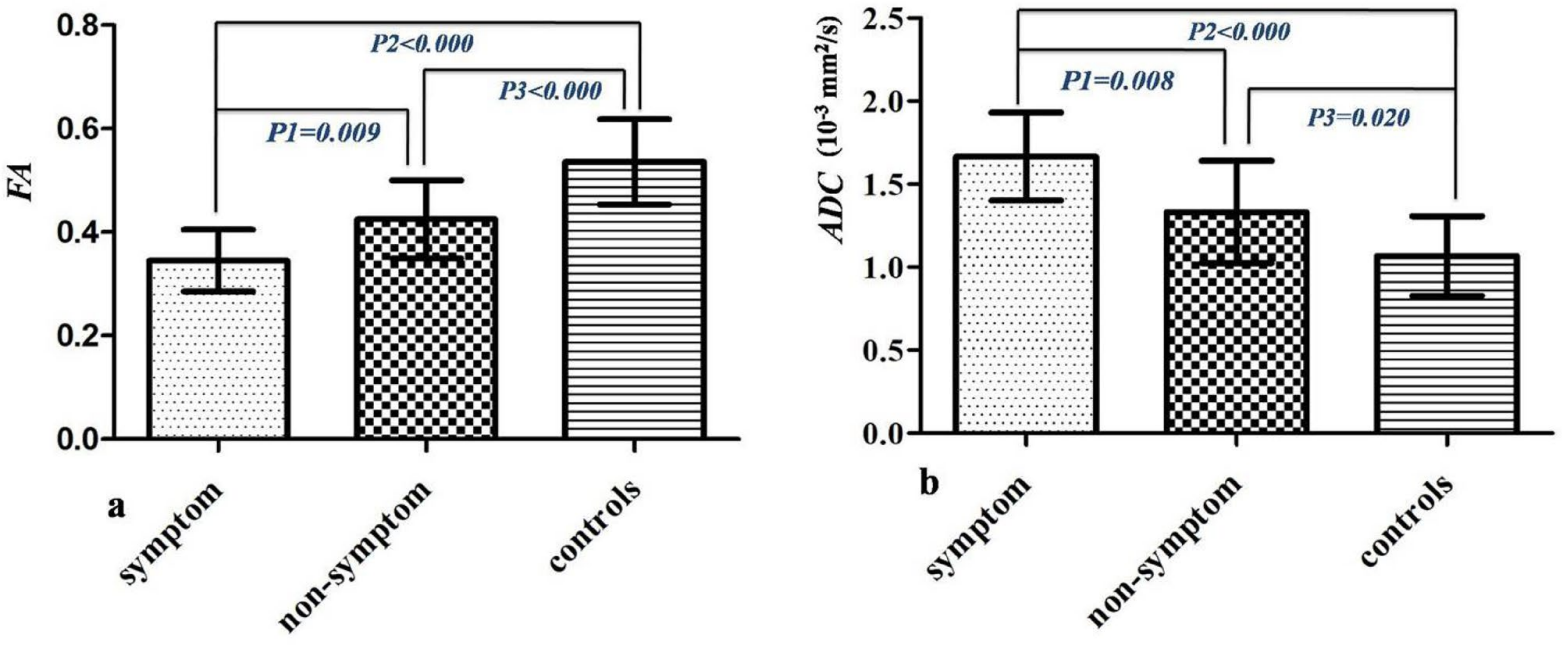
The differences of DTI parameters among patients with clinical symptom, patients without clinical symptom and normal controls. (**a**) The difference of FA values (mean + SD), (**b**) the difference of ADC values (mean + SD).

There were 9 patients in group A and 21 patients in group B. Two patients had muscle weakness and hypoesthesia, five patients had hypersensitivity or hypoesthesia, and two patients had shoulder and back pain. In group A, the FA and ADC values were 0.345 and 1.665 (mm^2^/s) at the maximal syrinx diameter, respectively. In group B, the FA value was 0.425 and the ADC value was 1.332 (mm^2^/s) at the maximal syrinx diameter/spinal cord diameter. FA values of group A were significantly lower than those of group B (*P* = 0.009) and controls (*P* < 0.000), while FA values of group B were notably lower than those of controls (*P* < 0.000) (Fig. 5a). The ADC values of group A were significantly higher than those of group B (*P* = 0.008) and controls (*P* < 0.000), and ADC values of group B were notably higher than those of controls (*P* = 0.020) (Fig. 5b).

## Discussion

Using DTI techniques in the spinal cord is crucial for discerning specific fibre tracts. This application could provide a particular assessment for identifying damage of spinal tracts as well as a better understanding of how tissue damage causes clinical deficits and why clinical symptoms can be present prior to neuro-radiological imaging^6,8,13^. In clinical practice, syringomyelia does not need to be treated for patients with no neurological symptoms or signs, even if the syringomyelia is very large. However, in some patients without clinical symptoms, the microstructural function of the spinal cord at the cavity may have been seriously disturbed, which requires timely intervention and treatment. DTI may be used in syringomyelia to investigate microstructural changes in spinal fibre tracts around and beyond the syrinx, which are not available in conventional MRI and other technologies^10,11,14^.

Currently, few studies have reported the application of DTI technology in patients with syringomyelia. In a study focusing on cervical spinal cord DTI and FT of patients with spinal cord cavities^15^, the authors found that FA values of patients with spinal cord was significantly lower than those of the normal population. However, there was no significant difference in ADC values between the patients with spinal cord cavities and normal controls. The study also found that in patients with spinal cord cavities, the cervical spinal cord segment with reduced FA values was highly consistent with a lack of neurological function and skin cold sensory abnormalities; additionally, there was a significant negative correlation between the FA value and the evoked potential value. Yan et al.^10^ found that the FA values were significantly reduced at the level of the syrinx in patients with Chiari I malformation and syringomyelia compared to the controls, whereas no significant difference in FA values was found in the spinal cord rostral and caudal to the syrinx in patients and controls. The study also showed that the FA values at the level of the syrinx were decreased significantly in symptomatic patients compared with non-symptomatic patients or the control group, and the non-symptomatic patients and controls were significantly different. An analysis of six patients with cervical syringomyelia and two controls conducted by Roser et al. found that FA values were lower at the level of all the syrinxes examined (0.19) and reached normal values beyond the syrinxes (0.53)^11^. However, the study sample was small, especially the controls. Moreover, the authors did not calculate the FA value of different cervical segments. In a study of 37 patients with syringomyelia and 21 controls, Hatem et al^16^ found that FA values of patients both with pain or without pain were significantly lower compared with controls, but no notable difference in ADC values between patients and controls was found. In addition, the authors concluded that the FA parameter might be a better DTI parameter to evaluate the microstructural damage of the spinal cord. In an animal study of spinal cavity after spinal cord trauma, Zhang et al.^14^ found that the FA values were significantly decreased and the ADC values were significantly increased after spinal cord injury. However, following repair of the spinal cord injury, even if a spinal cavity appeared, the FA values increased notably; additionally, there was no obvious change in the ADC values. Saksena et al.^13^ performed spinal DTI scans in four patients with cavities and found that FA values at the level of the cavity were considerably lower compared to normal controls; however, the ADC values were higher. Moreover, the authors found that the DTI parameters of the spinal cord above or below the cavity level were not significantly different from the normal population. Roser et al.^11^ performed DTI scans in six patients with small spinal cavities and found that the FA values at the spinal cavity level were reduced significantly compared to normal spinal cord. While the FA values of the spinal cord around the spinal cavity were not significantly different from those of a normal spinal cord, the electrophysiological results were closely associated with the decrease in FA values.

In the present study, DTI performed in 30 patients with syringomyelia demonstrated that preserved fibre tracts above and below the syrinx were integral. In patients with syringomyelia, the FA values were considerably lower and the ADC values were significantly decreased at all levels of the examined syrinx (C1–C7) but had normal values outside the syrinx area. The FA values of patients with clinical symptoms was significantly lower than those of patients without clinical symptoms and normal control participants, while the ADC values in patients with clinical symptoms were significantly higher than those of patients without clinical symptoms and normal participants.

The mechanism of the increase in the ADC value is not clear. ADC values reflect the ability of water molecules to diffuse into the specific tissue. A high ADC value suggests an increase in the Brownian movement of water molecules, indicating that the integrity of the specific tissue microstructure is reduced^8,17^. In the present study, ADC values of patients at all segments of the syrinx were higher compared to control values. The cause for the elevated ADC values is uncertain, however. Compression of the syrinx to spinal cord may cause ischemia, oedema, atrophy, anoxemia and cellular membrane injury which could increase cellular membrane permeability and disrupt the movement of water molecules as well as decrease perfusion^8,17,18^. Moreover, long-term compression of the spinal cord may cause the syrinx to enlarge, and water diffusivity along fibre tract route slows further that cannot be displayed on conventional MR examination. As the results of this study indicated, there was a positive association between the size of the syrinx cavity and ADC values. The results also showed that at all cervical segments, the ADC values outside of the visible syrinx were lower compared with ADC values at segments of the syrinx, although the values at C6 and C7 were not statistically different. Moreover, the ADC values outside of the visible syrinx were similar to those of controls. The results suggest that the ability of water molecules to diffuse and the composition of the tissue outside of the syrinx were not disturbed; additionally, the microstructure was integral. However, the integrity of the spinal cord at segments of the syrinx was damaged.

The FA value is typically close to 1.0 in intact neurons. When any damage to the axonal membrane occurs, the diffusion of water molecular at that level becomes unrestricted and isotropic^8^. In patients with syringomyelia, the present study showed that the FA values were reduced in syrinx segments. These results indicated that the diffusion of water molecule around the syrinx was restricted. The reduction may be associated with intramedullary oedema and low fibre density, which caused an imbalance between intra- and extra-cellular spaces^17,18^. The present results also found that FA values outside syrinx areas were normal, which demonstrated that the microstructure of the spinal cord above and below segments of the syrinx was normal. Moreover, a bivariate analysis revealed a negative correlation between the size of the syrinx cavity and the FA values, suggesting that, at affected levels, the bigger the syrinx, the more serious the dysfunction caused by the unrestricted and isotropic diffusion of water molecules.

Previous studies found FA values were from 0.45 to 0.83 in the cervical spine of a normal, healthy population^19,20^. A study including 20 normal participants concluded that FA values at different cervical cord levels were significantly different. However, ADC values between the levels were not significantly different^6^. In addition, some studies have showed that DTI parameters significantly differed at different cervical spinal cord levels^5,19,20^. However, another study demonstrated no obvious differences in cervical cord levels^7^. The present results indicated that both FA values and ADC values were not significantly different among normal cervical levels. Therefore, DTI parameters of different sections of cervical cord should be investigated further for comparison.

There were several limitations to the present study. First, the study did not examine the correlation between electrophysiological values and DTI parameters. Second, the control sample was small. Third, the present study only analysed the DTI values in the cervical spinal cord; further studies will focus on differences in DTI parameters in the thoracic spinal cord in patients with syringomyelia.

## Conclusions

Our preliminary results in this small sample support a decrease of FA values and an increase of ADC values in the syrinx cavity compared to controls. FA values or ADC values at all cervical levels were not significantly different in both controls and outside of the syrinx. No significant differences in mean FA or ADC values were found outside the area of the visible syrinx and in controls. The association between the size of the syrinx cavity and FA values was negative, and ADC values was positive. The DTI parameters were different in patients with clinical symptoms compared to patients without clinical symptoms and healthy controls.

## Data Availability

All data generated or analysed during this study are included in this published article.
